# Thymic Neuroendocrine Tumor Presenting With Cervical Lymphadenopathy: A Case Report and Diagnostic Challenge

**DOI:** 10.1002/ccr3.71580

**Published:** 2025-12-12

**Authors:** Xiaohui Xie, Jinming Tang, Wenxiang Wang, Desong Yang, Kele Qin

**Affiliations:** ^1^ Department of Cardiovascular Surgery The Second Xiangya Hospital, Central South University Changsha China; ^2^ Second Department of Thoracic Surgery, Hunan Cancer Hospital/The Affiliated Cancer Hospital of Xiangya School of Medicine, Central South University Changsha China

**Keywords:** atypical carcinoid, capecitabine‐temozolomide, cervical lymphadenopathy, thymic neuroendocrine tumor

## Abstract

Thymic neuroendocrine tumors are rare and may initially present with isolated cervical lymphadenopathy. Early recognition and a multidisciplinary diagnostic approach are crucial for timely identification, accurate localization, and curative resection.

**Abbreviations**
CAPTEM: capecitabine and temozolomide
CT: computed tomography
ENETS: European Neuroendocrine Tumor Society
NCCN: National Comprehensive Cancer Network
NET G2: grade 2 neuroendocrine tumor
NETs: neuroendocrine tumors
NSE: neuron‐specific enolase
OS: overall survival
PET‐CT: positron emission tomography–computed tomography
PRRT: peptide receptor radionuclide therapy
R0 resection: complete resection with negative margins
SEER: Surveillance, Epidemiology, and End Results
SSTR2: somatostatin receptor subtype 2
WHO: World Health Organization

## 1 Introduction

Neuroendocrine tumors (NETs) comprise a heterogeneous and increasingly recognized group of neoplasms arising from neuroendocrine cells, which are ubiquitously distributed throughout the body, most frequently involving the gastrointestinal tract, pancreas, and lungs [1, 2]. These neoplasms are characterized by their capacity to synthesize, store, and secrete various peptides, amines, and neuroendocrine markers, potentially leading to distinct clinical syndromes, such as carcinoid syndrome, or remaining clinically silent for extended periods [2, 3]. Over recent decades, both the incidence and prevalence of NETs have risen markedly, attributable to improvements in diagnostic modalities and heightened clinical awareness [4]. Epidemiological data demonstrate that the age‐adjusted incidence of NETs in the United States has increased from 1.09 per 100,000 in 1973 to more than 6.98 per 100,000 in recent years, underscoring the growing clinical relevance of these tumors [1].

The clinical manifestations of NETs are remarkably heterogeneous, primarily influenced by the anatomical location, secretory activity, and extent of tumor. A substantial proportion of patients remain clinically silent until metastatic spread has occurred, at which juncture nonspecific manifestations such as lymphadenopathy, unexplained weight loss, or various paraneoplastic syndromes may emerge. Metastatic NETs of unknown primary origin represent a significant diagnostic challenge due to their intrinsic rarity, considerable histopathological overlap with other neoplastic entities, and frequent lack of site‐specific clinical signs [5, 6].

NETs display a broad spectrum of biological behavior, ranging from well‐differentiated, indolent neoplasms to poorly differentiated, highly aggressive carcinomas. The World Health Organization (WHO) classification has evolved to incorporate tumor grade, proliferative index, thereby establishing a standardized and clinically relevant framework to facilitate accurate diagnosis and guide therapeutic management [7]. Thymic and other extra‐gastrointestinal NETs, including those arising in the mediastinum, are exceedingly rare and typically present at advanced stages, frequently with metastasis to lymph nodes, liver, bone, or, as in this report, the cervical region [8, 9].

Recent advancements in diagnostic strategies, notably the advent of somatostatin receptor‐based positron emission tomography–computed tomography (PET‐CT), refined histopathological analyses, and molecular profiling, have significantly improved the localization and characterization of both primary and metastatic NETs [10]. Nevertheless, optimal management remains complex and necessitates a multidisciplinary approach involving oncology, pathology, radiology, surgery, and nuclear medicine.

Herein, we report a rare case of a metastatic neuroendocrine tumor presenting as cervical lymphadenopathy, with the primary site ultimately identified as the thymus. We discuss the diagnostic complexity and therapeutic considerations in light of recent developments in neuroendocrine tumor management.

## 2 Case History

A 44‐year‐old male patient presented in September 2023 with a self‐detected, asymptomatic mass located in the left cervical region. Initially, the patient did not pay attention to the mass until January 2024, when bilateral cervical lymphadenopathy was detected during a routine physical examination. The patient subsequently presented to the department of thoracic surgery at our hospital for further evaluation. On admission, clinical assessment demonstrated satisfactory nutritional status and general well‐being, with no signs of jaundice, rash, or bleeding diathesis. Physical examination revealed multiple enlarged, freely mobile lymph nodes in the cervical and supraclavicular regions, with the largest measuring approximately 2 cm in diameter. No other abnormalities were detected on systemic examination.

**FIGURE 1:**
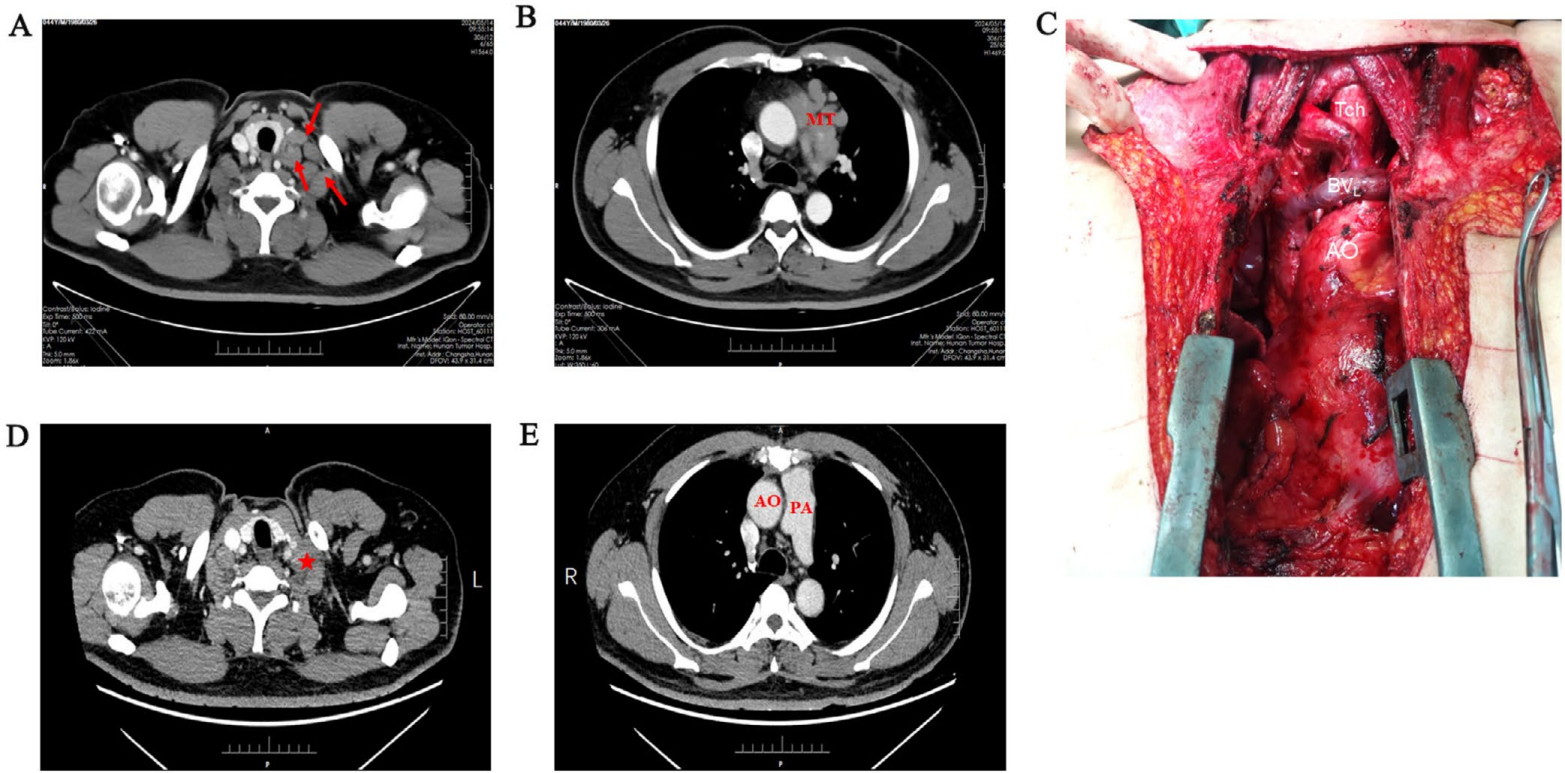
Preoperative and postoperative CT scan images and intraoperative anatomical illustration of the patient. (A) Axial CT image at the trachea‐thyroid level showed abnormal cervical lymph node clusters. Red arrows indicating enlarged lymph nodes. (B) Axial CT image at the carina level demonstrating a mediastinal tumor. MT, mediastinal tumor. (C) The surgery involved complete resection of the thymus containing the tumor and pericardial fat. Resection extended superiorly to the left brachiocephalic vein, inferiorly to the diaphragm, and laterally to the phrenic nerves, encompassing all perithymic and pericardial fat. AO, aorta; BV_L_, left brachiocephalic vein; Tch, trachea. (D, E) postoperative CT scan images at the trachea‐thyroid level showed.

On January 22, 2024, contrast‐enhanced computed tomography (CT) revealed a mediastinal mass with bilateral supraclavicular and right cervical lymphadenopathy (Figure 1A,B). Because PET‐CT using somatostatin‐receptor–targeted radiopharmaceuticals was unavailable locally, the patient underwent 18F‐fluorodeoxyglucose (18F‐FDG) PET‐CT. This study showed abnormal FDG uptake in the enlarged supraclavicular and right cervical lymph nodes, indicating a high likelihood of malignancy and prompting biopsy. Ultrasound‐guided core needle biopsy of the left cervical lymph node revealed metastatic neuroendocrine tumor, with histopathological features consistent with atypical carcinoid. Immunohistochemical analysis showed positivity for CD117, INSM1, SSTR2 and synaptophysin, with a Ki‐67 proliferation index of approximately 30%. The tumor cells were negative for CD5, S‐100, P40, TTF‐1, P63, CK5/6, CK7, calretinin, and Epstein–Barr virus‐encoded RNA. These findings support the diagnosis of metastatic, moderately differentiated neuroendocrine tumor.

## 3 Differential Diagnosis, Investigations and Treatment

The initial presentation of painless cervical lymphadenopathy necessitated a comprehensive differential diagnosis. Reactive lymphadenopathy secondary to viral infections, bacterial lymphadenitis, and granulomatous diseases such as tuberculosis or sarcoidosis were initially considered. Given the patient's age and the absence of systemic symptoms such as fever, night sweats, or weight loss, lymphoma, particularly Hodgkin lymphoma, emerged as a primary consideration. Metastatic squamous cell carcinoma originating from the head and neck region also remained a possibility. However, nasopharyngolaryngoscopic evaluation did not reveal any mucosal lesions. Furthermore, the absence of Epstein–Barr virus‐encoded RNA and negative immunohistochemical staining for markers including TTF‐1, CK5/6, and P40 effectively ruled out nasopharyngeal carcinoma and pulmonary squamous cell carcinoma. The detection of neuroendocrine markers, including synaptophysin, INSM1, and CD117, in conjunction with a high Ki‐67 proliferation index, redirected diagnostic suspicion toward metastatic neuroendocrine neoplasms. Subsequent PET‐CT imaging revealed a hypermetabolic mediastinal mass in the absence of pulmonary or gastrointestinal primary lesions, which ultimately supported the diagnosis of a thymic‐origin atypical carcinoid tumor.

From February to April 2024, the patient received three cycles of capecitabine plus temozolomide (CAPTEM) as neoadjuvant chemotherapy. We selected this regimen based on small series indicating that CAPTEM can downsize well‐differentiated neuroendocrine tumors and facilitate complete resection. Because repeat ^18^F‐FDG PET/CT was not available after CAPTEM, treatment response was assessed by contrast‐enhanced CT, which showed stable mediastinal disease by Response Evaluation Criteria in Solid Tumors (RECIST) and no new distant metastases. After multidisciplinary evaluation confirmed surgical eligibility and informed consent was obtained, the patient underwent median sternotomy with resection of the mediastinal mass and thymus, as well as bilateral cervical lymph node dissection, on May 22, 2024. Intraoperatively, involvement of the pericardium by tumor was observed, requiring partial pericardiectomy. The surgical procedure was completed uneventfully, achieving complete resection of the tumor and cervical lymph nodes (Figure 1C).

## 4 Molecular Profiling and Somatostatin Receptor Evaluation

Molecular characterization is increasingly recognized as an important component of neuroendocrine tumor management. After confirming the diagnosis of a thymic‐origin neuroendocrine tumor, we assessed somatostatin receptor subtype 2 (SSTR2) expression by immunohistochemistry. The tumor displayed moderate membranous staining in more than half of the cells, consistent with reports that approximately 57% of thymic neuroendocrine tumors express SSTR2 and that strong membrane expression is seen in roughly one quarter of cases [11]. Because expression of somatostatin receptors is a prerequisite for peptide receptor radionuclide therapy (PRRT), these results suggest that the patient could be a candidate for SSTR‐targeted imaging and therapy in the event of disease progression.

*MEN1* mutations underlie multiple endocrine neoplasia type 1 and constitute frequent somatic alterations in sporadic pancreatic neuroendocrine tumors [12]. *GTF2I* hotspot mutations are characteristic of thymic epithelial tumors rather than thymic NETs [13]. In light of these etiologic distinctions relative to thymic NETs, the absence of a family history of endocrine neoplasia, and the generally low mutational burden reported in thymic NETs, as well as the unavailability of a targeted gene panel at our institution at the time of care, we did not pursue comprehensive genomic sequencing.

## 5 Conclusion and Results

**FIGURE 2:**
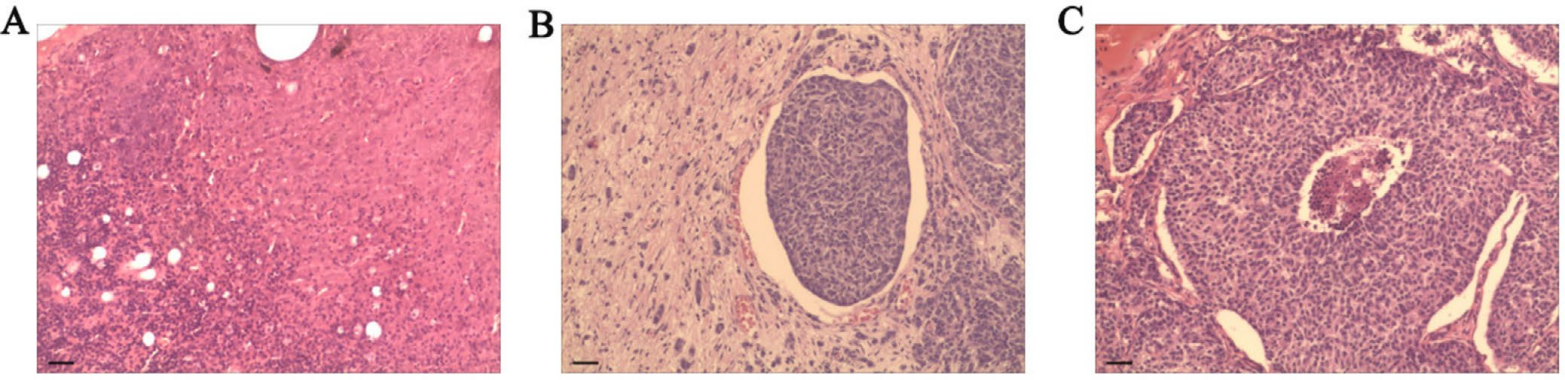
Postoperative pathology of the patient diagnosed with atypical Carcinoid (Neuroendocrine Tumor G2). (A) Hematoxylin and eosin (H&E) image showed scattered infiltrates of tumor cells within the background tissue. Focal areas of solid tumor growth are observed, providing an overview of the spatial distribution of neoplastic cells. Bar: 100 μm (B) H&E staining reveals a well‐circumscribed nest of tumor cells separated by fibrous stroma. Vascular tumor thrombi are identified. The tumor cells exhibit moderate nuclear atypia and granular chromatin. Bar: 50 μm (C) H&E staining demonstrates solid nests of uniform tumor cells with moderate to high cellularity. The central of mass show central necrosis. The tumor cells display round to oval nuclei with characteristic salt‐and‐pepper chromatin, typical of an atypical carcinoid tumor. Bar: 50 μm.

Histopathological examination of the resected specimens confirmed metastatic involvement in multiple enlarged and firm cervical lymph nodes, while no tumor infiltration was identified in the mediastinal lymph nodes, consistent with a complete resection with negative margins (R0 resection). Postoperative histopathological examination revealed a neoplastic lesion characterized by diffusely distributed tumor cells demonstrating focal infiltration and moderate nuclear atypia, along with frequent mitotic figures (Figure 2A). Numerous vascular tumor thrombi were identified (Figure 2B). Prominent fibrous septation and central necrosis were evident within the tumor mass (Figure 2C). In conjunction with immunohistochemical findings, these features were consistent with a grade 2 neuroendocrine tumor (NET G2). The patient had an uneventful postoperative recovery, with no significant discomfort. Follow‐up CT scans demonstrated complete removal of the mediastinal tumor and involved lymph nodes, with clear visualization of the aorta and pulmonary artery (Figure 1D,E).

Thymic‐origin neuroendocrine carcinoma remains a rare and challenging entity. Continued advancements in diagnostic imaging, molecular pathology, and therapeutic strategies are crucial. This case underscores the importance of a thorough and multidisciplinary approach to diagnosis and treatment, contributing to the growing body of knowledge and improving care for patients with this rare malignancy.

## 6 Postoperative Surveillance and Follow‐Up Plan

Given the high recurrence risk of thymic neuroendocrine tumors, we implemented a structured surveillance plan aligned with international guidance. The European Neuroendocrine Tumor Society (ENETS) recommends clinical review, cross‐sectional imaging, and laboratory testing every 3–6 months for 2–3 years after R0 resection, then every 6–12 months to 5 years [14]. The National Comprehensive Cancer Network (NCCN) advises imaging every 12 weeks in the first year, then every 6 months thereafter [15]. Accordingly, we scheduled visits every 4 months for 2 years, alternating chest/abdominal/pelvic CT or MRI with somatostatin receptor PET‐CT when available, with serum chromogranin A and neuron‐specific enolase measured at each visit. After 24 months, imaging will occur every 6 months for three additional years to enable early, potentially treatable detection of relapse.

Thymic‐origin neuroendocrine carcinoma remains rare and challenging. Ongoing advances in imaging, molecular pathology, and therapeutics are essential. This case underscores the value of multidisciplinary care and systematic follow‐up in optimizing outcomes for this uncommon malignancy.

## 7 Discussion

NETs of the thymus are exceedingly rare, accounting for less than 5% of all neuroendocrine carcinomas [9]. Thymic NETs, including atypical carcinoids, frequently present significant diagnostic and therapeutic challenges owing to their low incidence and propensity for late‐stage detection, often accompanied by metastatic disease. The present case illustrates these complexities, as the patient initially manifested with cervical lymphadenopathy rather than classic thoracic symptoms, highlighting the potential for atypical presentations that may lead to diagnostic delays.

The clinical manifestations of thymic NETs are frequently nonspecific, often mimicking other mediastinal or lymphoproliferative disorders. Such ambiguity may contribute to diagnostic delays, as exemplified in this patient. Advances in imaging modalities, particularly PET‐CT with radiolabeled somatostatin analogs, have substantially improved both the detection and localization of NETs, including those originating from the thymus [10, 16]. In this case, PET‐CT was pivotal in delineating the extent of disease and informing surgical planning. Furthermore, immunohistochemistry remains indispensable for establishing a definitive diagnosis and tumor grading, with markers such as CD117, INSM1, and Ki‐67 providing critical prognostic information.

Given the rarity of thymic NETs, treatment strategies are often extrapolated from guidelines for NETs arising at other anatomical sites [5]. Complete surgical resection with negative margins (R0 resection) remains the cornerstone of management for localized and resectable disease, offering the best chance for prolonged disease‐free survival [17]. In this patient, radical resection was accomplished through comprehensive lymphadenectomy and mediastinal tumor excision. The pivotal role of surgery is further underscored by the high risk of recurrence and limited efficacy of systemic therapies in advanced stages. Adjuvant therapies, such as chemotherapy with CAPTEM, may offer disease stabilization, particularly in cases with residual or metastatic disease, as demonstrated in this report.

Novel therapeutic modalities, including targeted therapies and peptide receptor radionuclide therapy, are being actively investigated for patients with advanced or recurrent NETs who have limited response to conventional treatments [18, 19]. Early data suggest that these strategies may enhance disease control and survival outcomes, though further validation in prospective clinical trials is warranted. The establishment of dedicated centers and multidisciplinary tumor boards is critical for optimizing individualized management strategies, particularly for rare entities such as thymic NETs.

Adjuvant systemic therapy remains controversial. CAPTEM, as used in our patient, is supported by small series and retrospective studies [20]. Emerging targeted options include tyrosine kinase inhibitors (e.g., everolimus) and PRRT. PRRT employs radiolabeled somatostatin analogues such as ^177^Lu‐DOTATATE to deliver targeted radiation to tumors expressing somatostatin receptors. Eligibility criteria include uptake on somatostatin receptor imaging at least equal to normal hepatic uptake (Krenning score ≥ 2), Karnofsky performance status ≥ 60 and adequate renal and hematologic function, whereas contraindications include pregnancy, severe cardiac impairment and life expectancy less than 3 months [21]. Although immunohistochemistry demonstrated SSTR2 positivity, somatostatin receptor imaging was not performed because ^68^Ga‐DOTA‐SSA PET‐CT was not readily accessible domestically and, routine ^18^F‐FDG PET‐CT served as the primary imaging modality. Provided that subsequent imaging demonstrates adequate somatostatin receptor expression and the disease exhibits recurrence or progression, PRRT may constitute a rational therapeutic approach. Early data suggest that PRRT and other targeted therapies can enhance disease control and survival outcomes, though further validation in prospective clinical trials is warranted [22].

Although the benefits of adjuvant therapy remain debated, evidence for neoadjuvant systemic therapy is even scarcer. After reviewing limited data from other NET subtypes, we decided to administer CAPTEM preoperatively. A 2024 case report of an atypical lung carcinoid treated with neoadjuvant CAPTEM demonstrated that 6 months of therapy yielded a partial response in mediastinal lymph nodes and enabled complete resection; the patient remained disease‐free 24 months postoperatively [23]. In addition, a review of neoadjuvant therapy across neuroendocrine neoplasms found that CAPTEM administered to six patients with borderline‐resectable pancreatic NETs resulted in tumor regression or stabilization in every case and allowed surgery with negative margins in four cases [24]. Although these reports address pancreatic and pulmonary rather than thymic NETs, they suggest that CAPTEM can downstage disease and improve resectability. In our case, CAPTEM maintained the stability of the mediastinal lesion until surgery.

Surgical management in this case involved partial pericardiectomy, necessitated by direct tumor invasion of the pericardium. Resection in proximity to vital mediastinal structures presents significant technical challenges; however, meticulous surgical planning enabled successful removal without major complications, such as recurrent laryngeal nerve injury. Preservation of normal postoperative vocal function attests to the importance of surgical expertise in achieving optimal outcomes. This case reinforces that, even in rare thoracic tumors involving critical anatomy, complete resection is feasible and can be accomplished safely in experienced centers.

NETs are rare neoplasms that generally have worse long‐term outcomes compared with other thymic tumors [25]. Large registry studies have confirmed this finding. An analysis of the Surveillance, Epidemiology, and End Results (SEER) database reported a median overall survival (OS) of approximately 6 years for patients with thymic NETs, and survival was significantly prolonged in those who underwent surgical resection, with a median OS of about 109 months compared with 46 months in patients who did not undergo surgery [17]. Similarly, recent single‐institution series have further demonstrated the prognostic challenges. One cohort reported three‐ and five‐year overall survival rates of approximately 75% and 70%, respectively, whereas only about 38% of patients remained progression‐free at 5 years, indicating a high recurrence rate despite active treatment [26]. In addition, several prognostic indicators have been identified across studies. A high tumor proliferative index (Ki‐67 > 10%, indicating higher histologic grade) and the absence of complete surgical resection are both significantly associated with poorer survival [27]. Furthermore, elevated serum neuroendocrine markers, such as neuron‐specific enolase (NSE), have been correlated with more aggressive disease and advanced stage at presentation [28]. Although this patient had a WHO grade 2 tumor and achieved complete (R0) resection, which suggests a relatively favorable prognosis, continued long‐term surveillance remains warranted. Despite growing international expertise in the management of rare NETs, including thymic subtypes, there remains an unmet need for larger, multicenter clinical trials and the development of evidence‐based, site‐specific guidelines. In China, increasing attention is being paid to the formulation of dedicated protocols and the advancement of specialized care for these uncommon malignancies. This case underscores the importance of international collaboration and robust clinical research to enhance understanding and improve prognostic outcomes for patients with rare thymic NETs.

## Author Contributions

**Xiaohui Xie:** writing – original draft, writing – review and editing. **Jinming Tang:** conceptualization, data curation. **Wenxiang Wang:** resources. **Desong Yang:** investigation, supervision. **Kele Qin:** investigation, supervision, validation, visualization.

## Funding

This work was financially supported by the Hunan Provincial Natural Science Foundation (2021JJ70103 to WW), Health Research Project of Hunan Provincial Health Commission (C2019074 to WW).

## Consent

All participants provided written informed consent for the use of their data and images in this publication.

## Conflicts of Interest

The authors declare no conflicts of interest.

## Data Availability

The datasets from the current study are available from the corresponding author upon reasonable request.

## References

[1] A. Dasari , C. Shen , D. Halperin , et al., “Trends in the Incidence, Prevalence, and Survival Outcomes in Patients With Neuroendocrine Tumors in the United States,” JAMA Oncology 3, no. 10 (2017): 1335–1342. 28448665 10.1001/jamaoncol.2017.0589 PMC5824320

[2] J. C. Yao , M. Hassan , A. Phan , et al., “One Hundred Years After “Carcinoid”: Epidemiology of and Prognostic Factors for Neuroendocrine Tumors in 35,825 Cases in the United States,” Journal of Clinical Oncology 26, no. 18 (2008): 3063–3072. 18565894 10.1200/JCO.2007.15.4377

[3] M. E. Caplin , E. Baudin , P. Ferolla , et al., “Pulmonary Neuroendocrine (Carcinoid) Tumors: European Neuroendocrine Tumor Society Expert Consensus and Recommendations for Best Practice for Typical and Atypical Pulmonary Carcinoids,” Annals of Oncology 26, no. 8 (2015): 1604–1620. 25646366 10.1093/annonc/mdv041

[4] M. Pavel , K. Öberg , M. Falconi , et al., “Gastroenteropancreatic Neuroendocrine Neoplasms: ESMO Clinical Practice Guidelines for Diagnosis, Treatment and Follow‐Up,” Annals of Oncology 31, no. 7 (2020): 844–860. 32272208 10.1016/j.annonc.2020.03.304

[5] M. Pavel , D. O'toole , F. Costa , et al., “ENETS Consensus Guidelines Update for the Management of Distant Metastatic Disease of Intestinal, Pancreatic, Bronchial Neuroendocrine Neoplasms (NEN) and NEN of Unknown Primary Site,” Neuroendocrinology 103, no. 2 (2016): 172–185. 26731013 10.1159/000443167

[6] B. Oronsky , P. C. Ma , D. Morgensztern , and C. A. Carter , “Nothing but NET: A Review of Neuroendocrine Tumors and Carcinomas,” Neoplasia 19, no. 12 (2017): 991–1002. 29091800 10.1016/j.neo.2017.09.002 PMC5678742

[7] G. Rindi , M. Falconi , C. Klersy , et al., “TNM Staging of Neoplasms of the Endocrine Pancreas: Results From a Large International Cohort Study,” Journal of the National Cancer Institute 104, no. 10 (2012): 764–777. 22525418 10.1093/jnci/djs208

[8] H. Bohnenberger , H. Dinter , A. König , et al., “Neuroendocrine Tumors of the Thymus and Mediastinum,” Journal of Thoracic Disease 9, no. Suppl 15 (2017): S1448–S1457. 29201448 10.21037/jtd.2017.02.02 PMC5690954

[9] Y.‐Z. Wang , G. Mayhall , L. B. Anthony , R. J. Campeau , J. P. Boudreaux , and E. A. Woltering , “Cervical and Upper Mediastinal Lymph Node Metastasis From Gastrointestinal and Pancreatic Neuroendocrine Tumors: True Incidence and Management,” Journal of the American College of Surgeons 214, no. 6 (2012): 1017–1022. 22521444 10.1016/j.jamcollsurg.2012.02.006

[10] V. Ambrosini , D. Campana , L. Bodei , et al., “68Ga‐DOTANOC PET/CT Clinical Impact in Patients With Neuroendocrine Tumors,” Journal of Nuclear Medicine 51, no. 5 (2010): 669–673. 20395323 10.2967/jnumed.109.071712

[11] A. C. Roden , S. Rakshit , G. B. Johnson , et al., “Correlation of Somatostatin Receptor 2 Expression, 68Ga‐DOTATATE PET Scan and Octreotide Treatment in Thymic Epithelial Tumors,” Frontiers in Oncology 12 (2022): 823667. 35198446 10.3389/fonc.2022.823667 PMC8859934

[12] C. D. Kamilaris and C. A. Stratakis , “Multiple Endocrine Neoplasia Type 1 (MEN1): An Update and the Significance of Early Genetic and Clinical Diagnosis,” Frontiers in Endocrinology 10 (2019): 339. 31263451 10.3389/fendo.2019.00339 PMC6584804

[13] Y. Feng , Y. Lei , X. Wu , et al., “GTF2I Mutation Frequently Occurs in More Indolent Thymic Epithelial Tumors and Predicts Better Prognosis,” Lung Cancer 110 (2017): 48–52. 28676218 10.1016/j.lungcan.2017.05.020

[14] R. Arnold , Y.‐J. Chen , F. Costa , et al., “ENETS Consensus Guidelines for the Standards of Care in Neuroendocrine Tumors: Follow‐Up and Documentation,” Neuroendocrinology 90, no. 2 (2009): 227–233. 19713715 10.1159/000225952

[15] J. A. Castellanos and N. B. Merchant , “Intensity of Follow‐Up After Pancreatic Cancer Resection,” Annals of Surgical Oncology 21, no. 3 (2014): 747–751. 24092447 10.1245/s10434-013-3289-7 PMC4066734

[16] T. A. Hope , E. K. Bergsland , M. F. Bozkurt , et al., “Appropriate Use Criteria for Somatostatin Receptor PET Imaging in Neuroendocrine Tumors,” Journal of Nuclear Medicine 59, no. 1 (2018): 66–74. 29025982 10.2967/jnumed.117.202275 PMC6910630

[17] P. Gaur , C. Leary , and J. C. Yao , “Thymic Neuroendocrine Tumors: A SEER Database Analysis of 160 Patients,” Annals of Surgery 251, no. 6 (2010): 1117–1121. 20485130 10.1097/SLA.0b013e3181dd4ec4

[18] J. Strosberg , G. El‐Haddad , E. Wolin , et al., “Phase 3 Trial of 177Lu‐Dotatate for Midgut Neuroendocrine Tumors,” New England Journal of Medicine 376, no. 2 (2017): 125–135. 28076709 10.1056/NEJMoa1607427 PMC5895095

[19] K. Öberg , U. Knigge , D. Kwekkeboom , et al., “Neuroendocrine Gastro‐Entero‐Pancreatic Tumors: ESMO Clinical Practice Guidelines for Diagnosis, Treatment and Follow‐Up,” Annals of Oncology 23, no. Suppl 7 (2012): vii124–vii130. 22997445 10.1093/annonc/mds295

[20] X. Wang , Y. Li , J. Duan , et al., “Capecitabine and Temozolomide as a Promising Therapy for Advanced Thymic Atypical Carcinoid,” Oncologist 24, no. 6 (2019): 798–802. 30413666 10.1634/theoncologist.2018-0291 PMC6656479

[21] V. Ambrosini , L. Zanoni , A. Filice , et al., “Radiolabeled Somatostatin Analogues for Diagnosis and Treatment of Neuroendocrine Tumors,” Cancers (Basel) 14, no. 4 (2022): 1055. 35205805 10.3390/cancers14041055 PMC8870358

[22] G. Kong , S. Grozinsky‐Glasberg , M. S. Hofman , et al., “Efficacy of Peptide Receptor Radionuclide Therapy for Functional Metastatic Paraganglioma and Pheochromocytoma,” Journal of Clinical Endocrinology and Metabolism 102, no. 9 (2017): 3278–3287. 28605448 10.1210/jc.2017-00816

[23] G. Evangelou , I. Vamvakaris , I. Konstantopoulou , and K. Syrigos , “Neoadjuvant Capecitabine Plus Temozolomide in Atypical Lung NETs,” Oncology (Williston Park, N.Y.) 38, no. 7 (2024): 264–268. 39024198 10.46883/2024.25921024

[24] A. Lania , F. Ferraù , M. Rubino , R. Modica , A. Colao , and A. Faggiano , “Neoadjuvant Therapy for Neuroendocrine Neoplasms: Recent Progresses and Future Approaches,” Frontiers in Endocrinology 12 (2021): 651438. 34381421 10.3389/fendo.2021.651438 PMC8350565

[25] P. L. Filosso , X. Yao , U. Ahmad , et al., “Outcome of Primary Neuroendocrine Tumors of the Thymus: A Joint Analysis of the International Thymic Malignancy Interest Group and the European Society of Thoracic Surgeons Databases,” Journal of Thoracic and Cardiovascular Surgery 149, no. 1 (2015): 103–109. 25308116 10.1016/j.jtcvs.2014.08.061

[26] C. A. Moran and S. Suster , “Neuroendocrine Carcinomas (Carcinoid Tumor) of the Thymus. A Clinicopathologic Analysis of 80 Cases,” American Journal of Clinical Pathology 114, no. 1 (2000): 100–110. 10884805 10.1309/3PDN-PMT5-EQTM-H0CD

[27] G. Cardillo , F. Rea , M. Lucchi , et al., “Primary Neuroendocrine Tumors of the Thymus: A Multicenter Experience of 35 Patients,” Annals of Thoracic Surgery 94, no. 1 (2012): 241–246. 22632882 10.1016/j.athoracsur.2012.03.062

[28] Z. Cheng , F. Yu , R. Chen , et al., “Treatment, Prognostic Markers, and Survival in Thymic Neuroendocrine Tumors, With Special Reference to Temozolomide‐Based Chemotherapy,” Cancers (Basel) 16, no. 14 (2024): 2502. 39061142 10.3390/cancers16142502 PMC11275075

