# Correction to “Thymic Neuroendocrine Tumor Presenting with Cervical Lymphadenopathy: A Case Report and Diagnostic Challenge”

**DOI:** 10.1002/ccr3.72063

**Published:** 2026-02-23

X. Xie, J. Tang, W. Wang, D. Yang, and K. Qin, “Thymic Neuroendocrine Tumor Presenting With Cervical Lymphadenopathy: A Case Report and Diagnostic Challenge,” *Clinical Case Reports* 13, no. 12 (2025): e71580, https://doi.org/10.1002/ccr3.71580.

The order of the affiliations to article being corrected.

The order of the institutions in the “Author Affiliations” section of this article is incorrect. The correct order of the institutions should be as follows:

1. Second Department of Thoracic Surgery, Hunan Cancer Hospital/The Affiliated Cancer Hospital of Xiangya School of Medicine, Central South University, Changsha, China.
2. Department of Cardiovascular Surgery, The Second Xiangya Hospital, Central South University, 139 Middle Renmin Road, Changsha 410011, China

We apologize for this error.

